# Implementation of a Co-Design Strategy to Develop a Dashboard to Support Shared Decision Making in Advanced Cancer and Chronic Kidney Disease

**DOI:** 10.3390/jcm13144178

**Published:** 2024-07-17

**Authors:** Victoria Morken, Laura M. Perry, Ava Coughlin, Mary O’Connor, Ryan Chmiel, Stavroula Xinos, John Devin Peipert, Sofia F. Garcia, Jeffrey A. Linder, Ronald T. Ackermann, Sheetal Kircher, Nisha A. Mohindra, Vikram Aggarwal, Melissa Weitzel, Eugene C. Nelson, Glyn Elwyn, Aricca D. Van Citters, Cynthia Barnard, David Cella, Lisa R. Hirschhorn

**Affiliations:** 1 Department of Medical Social Sciences, Northwestern University Feinberg School of Medicine, 625 N Michigan Ave., Suite 2100, Chicago, IL 60611, USA; victoria.morken@northwestern.edu (V.M.); laura.perry@northwestern.edu (L.M.P.); ava.coughlin@northwestern.edu (A.C.); mary.oconnor@northwestern.edu (M.O.); sofia-garcia@northwestern.edu (S.F.G.); 2 Robert H. Lurie Comprehensive Cancer Center, Northwestern University Feinberg School of Medicine, 675 N St Clair St Fl 21 Ste 100, Chicago, IL 60611, USA; s-kircher@northwestern.edu (S.K.); d-cella@northwestern.edu (D.C.); 3 Northwestern Medicine, 251 E Huron St., Chicago, IL 60611, USA; rchmiel@nm.org (R.C.); sxino@nm.org (S.X.); 4 Department of Psychiatry and Behavioral Sciences, Northwestern University Feinberg School of Medicine, 425 E Ontario St #7, Chicago, IL 60611, USA; 5 Division of General Internal Medicine, Department of Medicine, Northwestern University Feinberg School of Medicine, 676 North St. Clair Street, Arkes Suite 2330, Chicago, IL 60611, USA; jlinder@northwestern.edu (J.A.L.); r.ackermann@northwestern.edu (R.T.A.); cbarnard@nm.org (C.B.); 6 Institute for Public Health and Medicine Northwestern University Feinberg School of Medicine, 420 E. Superior St. 6th Floor, Chicago, IL 60611, USA; 7 Division of Hematology and Oncology, Department of Medicine, Northwestern University Feinberg School of Medicine, 676 North St. Clair Street, Arkes Suite 2330, Chicago, IL 60611, USA; n-mohindra@northwestern.edu; 8 Division of Nephrology and Hypertension, Northwestern University Feinberg School of Medicine, 676 N St. Clair Street, Suite 2020, Chicago, IL 60611, USA; vikram.aggarwal@northwestern.edu (V.A.); mweitzel@nm.org (M.W.); 9 The Dartmouth Institute for Health Policy & Clinical Practice, Geisel School of Medicine at Dartmouth, 5 WTRB, Level 5, One Medical Center Drive, Lebanon, NH 03756, USA; eugene.c.nelson@gmail.com (E.C.N.); glynelwyn@gmail.com (G.E.); aricca.d.van.citters@dartmouth.edu (A.D.V.C.); 10 Robert J. Havey, MD Institute for Global Health, Northwestern University Feinberg School of Medicine, 259 E Erie St Ste 2350, Chicago, IL 60611, USA

**Keywords:** co-design, co-production, shared decision-making, implementation, patient-reported outcome measures, medical informatics, oncology, nephrology

## Abstract

**Background:** Shared decision making (SDM) is the process by which patients and clinicians exchange information and preferences to come to joint healthcare decisions. Clinical dashboards can support SDM by collecting, distilling, and presenting critical information, such as patient-reported outcomes (PROs), to be shared at points of care and in between appointments. We describe the implementation strategies and outcomes of a multistakeholder collaborative process known as “co-design” to develop a PRO-informed clinical dashboard to support SDM for patients with advanced cancer or chronic kidney disease (CKD). **Methods:** Across 14 sessions, two multidisciplinary teams comprising patients, care partners, clinicians, and other stakeholders iteratively co-designed an SDM dashboard for either advanced cancer (N = 25) or CKD (N = 24). Eligible patients, care partners, and frontline clinicians were identified by six physician champions. The co-design process included four key steps: (1) define “the problem”, (2) establish context of use, (3) build a consensus on design, and (4) define and test specifications. We also evaluated our success in implementing the co-design strategy using measures of fidelity, acceptability, adoption, feasibility, and effectiveness which were collected throughout the process. **Results:** Mean (*M*) scores across implementation measures of the co-design process were high, including observer-rated fidelity and adoption of co-design practices (*M* = 19.1 on a 7–21 scale, *N* = 36 ratings across 9 sessions), as well as acceptability based on the perceived degree of SDM that occurred during the co-design process (*M* = 10.4 on a 0 to 12 adapted collaboRATE scale). Capturing the feasibility and adoption of convening multistakeholder co-design teams, min–max normalized scores (ranging from 0 to 1) of stakeholder representation demonstrated that, on average, 95% of stakeholder types were represented for cancer sessions (*M* = 0.95) and 85% for CKD sessions (*M* = 0.85). The co-design process was rated as either “fully” or “partially” effective by 100% of respondents, in creating a dashboard that met its intended objective. **Conclusions:** A co-design process was successfully implemented to develop SDM clinical dashboards for advanced cancer and CKD care. We discuss key strategies and learnings from this process that may aid others in the development and uptake of patient-centered healthcare innovations.

## 1. Background

Shared decision making (SDM) is an evidence-based intervention that involves a partnership through which clinicians and patients share health information and make treatment decisions together [1,2]. Shared decision making (SDM) has the potential to advance the delivery of high-value, patient-centered care. The National Quality Forum’s (NQF) 2020 report, The Care We Need: Driving Better Outcomes for People and Communities, included educating and engaging patients in healthcare decisions among five “foundational opportunities” to drive value and create a personalized, population-driven healthcare delivery system [1]. This approach draws upon both the expertise of the clinician and the knowledge, preferences, and lived experience of the patient [3]. Evidence suggests that integrating the patient’s unique perspective into treatment planning through SDM enables patients and clinicians to “co-produce” health in ways that can drive value and innovation [4]. Co-producing health entails partnerships between patients, clinicians, and care partners across multiple collaborative health-related processes: (1) co-assessing health status, (2) co-designing optimal health services (e.g., collaboratively developing novel healthcare solutions, interventions, or tools), (3) co-planning an individual patient’s treatments (i.e., engaging in SDM), and (4) co-delivering those treatments and services (e.g., clinician management and patient self-management of health and treatments) [4,5]. SDM enables patients to make informed decisions about their care, balancing the benefits of treatment with the related risks and aligning with their priorities [1]. It has also been shown to reduce costs, improve health outcomes, and increase equity across patients [6]. These benefits can make a significant impact, particularly in the management of symptomatic, chronic diseases such as advanced cancer and chronic kidney disease [2].

However, there are considerable barriers to implementing SDM in clinical practice [7]. These include limited time in appointments, knowledge of how to effectively engage and inform patients, challenges associated with capturing patients’ needs and preferences for healthcare decision making, and organizational incentives for implementing SDM [8,9]. Difficulties may also exist with ensuring that patients understand their personal health data and the importance of patient-reported outcomes (PROs) so they can make informed decisions and actively participate in coproducing their health [3]. Thus, designing and implementing SDM tools into healthcare delivery systems is a needed strategy to optimize clinical encounters and drive value-based care.

Data-sharing tools (e.g., clinical dashboards) within the electronic health record (EHR) hold promise for encouraging the adoption of SDM in busy clinics serving patients with chronic conditions [5,10,11]. Patients and clinicians managing a chronic disease face a series of short- and long-term decisions that are often complicated by a sizeable amount of health information, multiple clinicians, and diverse patient needs and preferences. Collecting PROs in clinical practice can help to address these common challenges and inform shared treatment decisions for symptomatic chronic conditions such as cancer or CKD [12,13]. Furthermore, investigators have piloted the use of data visualization dashboards for use in chronic disease settings, such as cystic fibrosis [14,15,16], inflammatory bowel disease [13], arthritis [17,18], chronic kidney disease [19], and serious illness care [20]. Such dashboards combine clinical and patient-generated data, including PROs, into a single display within the EHR to facilitate discussion and SDM during appointments, and some are designed to help patients monitor their progress outside of appointments [13,14,17,20]. Ideally, patients and clinicians collaborate as partners to co-design tools intended to support the coproduction of healthcare through SDM [21]. Co-design is defined as the integration and involvement of the individuals who will use or be affected by a service or product in its design and has been shown to be effective in making these services or products more people-centered and reflective of their needs and priorities [22].

Implementation science offers methods to study evidence-based approaches to co-design and implement patient care tools such as an SDM dashboard. Implementation science was developed to accelerate the gap between evidence and implementation into practice of interventions found to be effective. The methods include measurement of implementation outcomes which measure the success in bringing the intervention into use as planned, such as co-designing a product [23]. In this paper, we describe the implementation process and outcomes (e.g., fidelity, feasibility, effectiveness) of using a multistakeholder co-design strategy to develop data visualization dashboards for co-producing healthcare via SDM in advanced cancer and CKD care [20]. This study focused on advanced cancer and CKD as examples of high-need conditions for using the dashboard based on their considerable symptom burden and high costs [24,25]. We hope this work will help to inform and improve the development and implementation of other evidence-based, patient-centered care approaches in the future.

## 2. Methods

All study procedures were approved by the Northwestern University Institutional Review Board, effective 25 February 2019 (STU00210091, STU00211654, and STU00212634).

### 2.1. Dashboard Co-Design Process

#### 2.1.1. Proposed SDM Dashboard and Setting

The investigators aimed to support the SDM process for advanced cancer and CKD by developing a data visualization dashboard that provides patients and clinicians with timely, desirable, and easy-to-interpret information about the patient’s health status and preferences. To make this information available for patients and clinicians at the point of care, the proposed dashboard would interface with the local EHR of a tertiary-care academic medical center in the midwestern United States. Therefore, the dashboard is built on the medical center’s existing infrastructure already embedded within the EHR, which routinely screens, scores, and generates clinical alerts for PROs in cancer care, and also expands the system to CKD care.

This existing infrastructure, called cPRO, is programmed to send automated invitations to patients 72 h before an upcoming oncology visit to complete five PROMIS symptom domains: depression, anxiety, fatigue, pain interference, and physical function. If patients score above pre-specified alert thresholds on any domain, clinicians receive automatic clinical alerts to inform supportive care efforts during the upcoming visit. For the present study, the proposed dashboard design process aimed to adapt the existing cPRO alert system into a centralized clinician- and patient-facing data visualization platform for SDM during routine visits for both cancer and CKD. To conceptualize the SDM dashboards for clinical use, including stakeholder-prioritized data elements to facilitate SDM during the visit, concurrent co-design activities were conducted in parallel for the two disease-specific groups: (1) those receiving specialty care for advanced lung or GI cancer and (2) those receiving specialty care and primary care for advanced CKD.

#### 2.1.2. Theoretical Model for Co-Production

From February 2019 to May 2020, we applied the Coproduction Design and Implementation Flow Model (CDIFM) [26,27] to conceptualize and co-design SDM dashboards for these two populations. This CDIFM is a user-centered engineering co-design framework that includes four key end-user activities: (1) define the problem, (2) establish context of use, (3) build consensus on design, and (4) define and test specifications [24,25]. The CDIFM has been successfully applied to the development of co-production dashboards in other chronic conditions and healthcare settings [13,14,17,20,28]. We collaborated with the authors of the CDIFM to devise a co-design strategy and five key principles to uphold during the process based on prior work [22,29]: (1) *inclusivity* (includes representatives from critical stakeholder groups), (2) *respectfulness* (all participants are seen as experts and their input is valued and has equal standing), (3) *participation* (uses dialogue and engagement to generate new, shared meanings based on expert knowledge and lived experience), (4) *iteration* (ideas and solutions are continually tested and evaluated; adaptations are a natural part of the process), and (5) *outcomes-focused* (designed to achieve an outcome or series of outcomes, where the potential solutions can be rapidly tested).

#### 2.1.3. Participants

Participants were chosen to represent the range of key stakeholders needed for co-design (see Table 1). For each disease group (advanced cancer [N = 25] and CKD [N = 24]), a team of stakeholders comprising patients, care partners (e.g., family members), physician champions, nurses, advanced practice professionals (APPs), other health professionals directly involved in care who might be using the dashboard during clinical interactions (e.g., social worker, dietician), and health information technology (HIT) specialists were assembled to engage in collaborative co-design. Physician participants identified a convenience sample of eligible patients under their care, their care partners, and other frontline clinicians. Eligible patients included those with a clinician-verified diagnosis of advanced cancer or CKD who were ≥18 years old, spoke English, and agreed to participate in the co-design process. As illustrated in Supplementary Materials File S1, patients from both codesign teams were present at and actively participated in every applicable working session. The research team identified HIT experts, researchers, and care quality representatives to participate in co-design sessions. Participation on the cancer and CKD teams was not mutually exclusive for clinical psychologists, HIT experts, health system quality leaders, and researchers. Members of the project team that planned and coordinated the sessions and research colleagues were assigned to each team to take notes and complete evaluations of sessions based on their field observations using a rubric described below (Supplementary Materials File S2).

#### 2.1.4. Dashboard Co-Design Process Cycle and Framework

**Figure 1:**
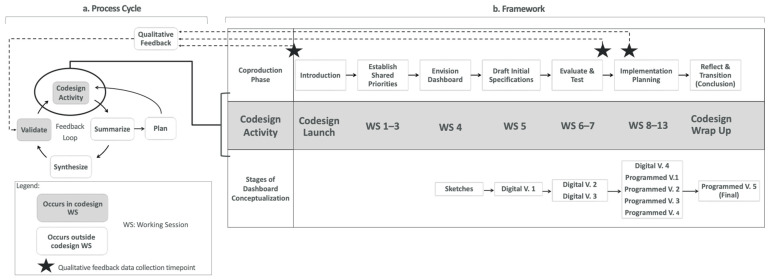
Dashboard co-design process cycle and framework. The co-design process cycle and framework depicts the cyclical feedback loop ((**a**) process cycle) that intersects with the linear, multi-phase framework (**b**) at each codesign activity (i.e., working sessions, launch and wrap up meetings) timepoint. The process cycle (**a**) illustrates the critical flow of actions surrounding each timepoint/codesign activity needed to advance through each phase of the framework (**b**) and drive conceptualization of the dashboard.

Based on the CDIFM, the study investigators developed a multi-step plan for dashboard co-design (Table 2) and co-design activities and oversaw the technical advancement of designs and supporting elements for the eventual translation of the dashboards. Figure 1 illustrates a cyclical process for planning and conducting each codesign activity (which includes a built-in feedback loop to inform subsequent sessions and adapt accordingly when change was needed) (Figure 1a) that maps to a linear framework (inspired by CDIFM) for dashboard co-design and conceptualization (see “co-production phase” in Figure 1b).

Figure 1a depicts the process cycle, including the following activities: Plan, Summarize, Synthesize, Validate, and (conduct) Codesign Activity. These concepts and their relationships are described below. First, codesign activities for each working session were outlined and mapped on to goals and deliverables for their respective phase of coproduction. After beginning the co-design process, planning for each working session was discrete and sequential, incorporating takeaways from prior sessions. Next, a codesign working session (i.e., “codesign activity”) was held where both teams would meet and work through their respective prompts of facilitated discussion and activities, while designated session observers took notes. After each session, analysis and summarization of session deliverables was completed by project staff to produce draft dashboard mockups and user flow diagrams, and to ensure that evolving ideas and designs were: (1) within the scope of the intended objectives, (2) representative of noted priorities across all stakeholder types, and (3) technically and logistically feasible. Additionally, insight gained from prior working sessions, including the teams’ challenges, strengths, and perceived adherence to SDM and codesign, were used to adaptively prepare plans for subsequent sessions. Careful synthesis of dashboard drafts and other products derived from session deliverables (along with new knowledge about the digital landscape and use environment) were fed back to the teams at succeeding sessions to validate that their needs and preferences were appropriately captured, and a tangible shared concept for teams to interact with, revise, and build from was provided.

The process framework (Figure 1b) depicts the linear phases of the dashboard co-design (i.e., the entire process from general concept to end product), where teams interactively engaged in the focused co-design activities relevant to each co-production phase: Introduction, Establish Shared Priorities, Envision Dashboard, Draft Initial Specifications, Evaluate and Test, Implementation Planning, and Reflect and Transition. Over time, these activities enabled our dynamic co-design teams to envision and develop end-product dashboards through an iterative process where dashboard designs evolved from low- to high-tech, starting with hand-drawn sketches and progressing to digital mockups and interactive programmed versions. Qualitative focus group data were also collected at three timepoints from a separate sample of stakeholder representatives of intended dashboard end users (i.e., patients, care partners, and clinicians) in advanced cancer and CKD to assess the feasibility, acceptability, and usability of the emerging products. The methods and outcomes of these focus groups will be published separately.

While Figure 1b categorizes each working session under one main coproduction phase (or correlated CDIFM concept), often, multiple CDIFM concepts were applied in any given session, with all CDIFM concepts linked to three or more individual co-design sessions in the process, as shown in Table 2 (column 5). For example, working session #4 was largely focused on the “envision the dashboard” concept, but also included elements related to “establishing shared priorities” and “defining specifications”, as teams were prompted to creatively consider what their dream dashboard could be after revisiting their prior work, discussing collective values and needs, while also starting to brainstorm potential specifications for the form and function of the future end product. This approach allowed teams to use the CDIFM concepts to ensure that the intended objectives of the program continued to be met as the dashboard development progressed over time via continuous comparison of the evolving designs against the original goals of the dashboard. Additionally, we convened a meeting to plan for clinic implementation with clinician champions and a final wrap-up meeting with all the stakeholders to share the pre-final dashboard and confirm fidelity to the dashboard goals. Team members also met monthly with Dartmouth collaborators to share their progress and to identify successes and address barriers.

Table 2 details the dashboard co-design framework, including activities, objectives, and outcomes of each session. Sessions were structured with pre-determined session objectives (i.e., goals for specific working session) and deliverables to promote teamwork, consistency, and concept advancement linked to specific components of the CDIFM (Table 2). We anticipated that there would be a fair amount of overlap in dashboard objectives across teams given the similarities in symptom burden and healthcare needs in advanced cancer and CKD. Furthermore, scalability to other diseases areas was an important factor in this work, and session objectives and deliverables were largely the same for both disease groups, driving the parallel development of two disease-specific dashboards to help the teams to understand cross-cutting concepts. Teams were given space to create independently from one another, recognizing that they would have disease-specific elements to explore and incorporate, as well as opportunities to share ideas between teams. This afforded both teams the benefit of learning from each other, gaining exposure to other ideas, and advocating for their own simultaneously. This helped both codesign teams streamline across their respective designs, generating dashboards with a similar look, feel, and functionality while maintaining the autonomy and technical flexibility to incorporate different data elements as was relevant per team (as determined by the teams’ patients, care partners, and clinicians).

Overarching team objectives (ultimate goals for their collaborative effort and intended end products) were directly developed by members of the co-design teams (i.e., patients, care partners, and clinicians) to establish team-driven priorities early in the co-design process for their respective diagnosis groups. Both codesign teams were given time and prompts to separately compose their objectives at working sessions #3 and #4. At this point in the process, they had built relationships within their respective teams and had shared personal experience with navigating CKD or advanced cancer management from their specific roles (i.e., clinician, patient, care partner). For example, team members collaboratively mapped the challenges they faced before, during, and after a clinical encounter. Clinicians were able to visualize and understand the experience from the patient’s perspective and vice versa. Teams were able to see what priorities were common across stakeholders and gained new understanding about why patients and clinicians sometimes wanted different information. The cancer team objective was “to co-design a dashboard that is a shared portal for oncology patients, care partners, and clinicians that will provide relevant, priority, and clear information in order to: optimize communication and collaboration, personalize care to patient needs, and improve quality, efficiency, and satisfaction”. The CKD team objective was “to provide quality and individualized kidney care by fostering trusting partnerships between patients, care partners, and care team by encouraging patient autonomy, engagement, and access to kidney health information in order to: help patients achieve their short-term and long-term goals during the management of CKD.’’ Session facilitators guided teams through activities and session processes, and outcomes were collected from completed individual or group worksheets, group debriefings prior to session closure, and facilitator notes, then synthesized after each session.

#### 2.1.5. Co-Design Strategies

We implemented several strategies to promote consistent, effective, and equitable stakeholder engagement guided by our five key principles of co-design (see Table 3). The dashboard design process included an iteration based on stakeholder feedback by following the CDIFM and continuing to revisit its concepts in consultation with our Dartmouth collaborators. Prototypes were presented at sessions to refine over time, validate final design, and inform dashboard programming needs. To remain outcome-focused during the sessions, we had pre-defined deliverables, and any ideas deemed outside the scope of the project were placed in a “parking lot” where they could be redirected in real time, as applicable, to other related initiatives. This strategy also fostered respect among team members and promoted continued sharing of ideas and refinement of the dashboard concept while still driving towards the end goal of dashboard development. Including HIT professionals with dedicated participation in the co-design process from start to finish was a strategy that facilitated the core principles of iteration and outcome-focused progress. The inclusion of these professionals allowed co-design teams to effectively translate their visions into tangible end products by ensuring that the dashboard features were feasible, efficient, and sustainable.

Equitable engagement and respect of voice were ensured by engaging all stakeholder types as equally valuable experts to mitigate any perceived power dynamics and establish a shared understanding that collective value of all perspectives was needed to co-design the dashboard. Furthermore, all codesign team participants were queried after working sessions to measure their individual perception of SDM at the session using the collaboRATE measure (see Section 2.2). Responses were regularly reviewed by session planners to intervene or adapt the session structure as needed (these data are outcome measures discussed in further detail below). We promoted inclusivity and participation of all people by involving them in setting a reasonable meeting schedule, providing appropriate incentives for participation, and including a flexible approach to active participation. For example, meetings started at 5:30 p.m. to accommodate work schedules, with dinner provided for all attendees, and stakeholders (clinicians, patients, and care partners) received a small stipend for every session attended. In-person attendance was encouraged, but options for remote attendance were offered to those who would otherwise be unable to participate. To ensure equal participation, some activities separated participants into smaller groups for more focused conversations, such as stakeholder-specific groups of patients and caregivers to discuss freely, without the influence of clinicians. In addition, groups were encouraged to welcome and explore differences in opinions to generate a deeper understanding of the shared values and individual priorities needed to make the dashboards both functional and valuable to all end users.

### 2.2. Co-Design Implementation Outcomes

We collected quantitative data from codesign sessions and team members to evaluate the success of our implementation of the co-design process. Data were collected from 4 sources: (1) operational and attendance logs kept for each co-design session, (2) observer rubrics for each co-design session, (3) post-session feedback surveys completed by team members after each co-design session, and (4) post-codesign reflection surveys that were completed by co-design team members after the co-design process was finished. We measured implementation outcomes based on the widely used Proctor’s definitions, including fidelity, acceptability, feasibility, adoption, and effectiveness [27] (Table 4).

#### 2.2.1. Fidelity to the Co-Design Process

To gather stakeholder perspectives on our fidelity to the five key principles of co-design, we presented co-design team members with a definition of each principle in the post-co-design reflection survey and asked them to rate how well each was met from 1 (not at all) to 4 (Extremely), for a total score ranging from 5 to 20. Based on the five key principles, we also derived an observer rubric of 7 practices that should occur at each co-design session. Outside observers who were not members of the co-design team attended each session and rated the adherence to each co-design practice from 1 (absent) to 3 (fulfilled), for a total session score ranging from 7 to 21 (see Table 4 and Supplementary Materials File S2 for the full measure).

#### 2.2.2. Fidelity to Shared Decision Making within Co-Design Process

Fidelity to SDM at each co-design session was self-reported by team members using an adaptation of the 3-item collaboRATE measure adapted for collaboration in the co-design process (see Table 4 for individual items) [14]. Total scores (ranging from 0 to 12) were rated after each co-design session.

#### 2.2.3. Acceptability

Acceptability was defined as participants’ satisfaction with the degree of SDM that occurred during the co-design process as a whole. Accordingly, the same 3-item adapted collaboRATE measure described above was included in the post-co-design reflection survey and rated by team members after the entire process was over.

#### 2.2.4. Feasibility

Feasibility was defined as the ability to obtain a full representation of stakeholders at each session, including patients, care partners, frontline clinicians, physician champions, HIT personnel, healthcare system quality personnel, and research personnel. This was measured by assigning 0 (not present) or 1 (present) to each type of stakeholder representative and then computing the proportion of total stakeholder types that were represented at each meeting, providing a min–max normalized score of stakeholder representation. In-person or remote attendance was also recorded, and the proportions were calculated per session (Supplementary Materials File S1).

#### 2.2.5. Adoption

Adoption was defined as the degree to which team members participated in the co-design activities. The same observer rubrics described above (“Fidelity to Co-design Process”) were used to reflect the adoption of the core co-design practices. In addition, the measure of stakeholder representation used to measure feasibility was also used to reflect the adoption of the multistakeholder co-design process.

#### 2.2.6. Effectiveness

The effectiveness of the process overall was measured by polling participants on the perceived achievement of their respective teams’ objectives after session 13. Participants responded to the question ‘’Does the dashboard meet the intended objective?” with either “Yes”, “Partially”, or “No”. Agreement with the question was defined as a response of either “Yes” or “Partially”.

### 2.3. Data Analysis

We calculated descriptive statistics, including range, mean scores, and standard deviations, across implementation measures (i.e., collaboRATE, observer rubric, and reflection survey). As a secondary analysis of the adapted collaboRATE surveys, we also analyzed the proportion of “top box” responses (a 12 out of 12 on the total score) for each session and averaged these proportions across all sessions [28]. To evaluate the fidelity and adoption of the process, post-session adapted collaboRATE surveys and observer rubrics were scored and then averaged across all sessions. To evaluate fidelity and acceptability, these constructs were also calculated from participants’ responses on relevant measures (i.e., self-report ratings of 5 key principles and adapted collaboRATE) in a post-co-design reflection survey after the co-design process was complete. For feasibility, mean values of the min–max normalized stakeholder representation scores across sessions were calculated. Attendance modality was analyzed according to the proportion of meetings for which each (live or remote) modality was used by at least one participant per co-design team. All implementation outcome measures are described in Table 3.

## 3. Results

### 3.1. Characteristics of Co-Design Meetings

Table 1 provides information on co-design team members’ roles and the number of participants in each role across the cancer team (n = 25), the CKD team (n = 24), and the total sample of participants (N = 35 due to participant overlap in the two teams; see Table 1). Co-design teams (cancer and CKD) together comprised a total of five patients, two patient care partners, eight frontline clinicians, six physicians, three HIT professionals, four health system quality leaders, and four researchers. Teams often met together and then separated to focus on disease-specific objectives or stakeholder-specific needs/preferences (patient/care partner vs. clinician) for either dashboard. Attendance of each stakeholder type across co-design working sessions can be found in Supplementary Materials File S1. General attendance was consistent throughout co-design. Attendance at each session ranged from 2 to 5 among patients, 1 to 2 among care partners, 3 to 7 among frontline clinicians, 2 to 5 among physician champions, 0 to 2 for HIT professionals, and 4 to 10 for facilitators (4 to 8 for project team members and 0 to 2 for quality leaders). The co-design process was completed with SDM dashboards in their pre-final stages of programming when the COVID-19 pandemic hit in March of 2020. All meetings held before the onset of the pandemic were attended in person by >90% of participants; one CKD patient had to attend remotely for every session, and there was very occasionally a second patient who had to attend remotely. The final co-design session “Wrap Up Meeting” was attended 100% remotely in May 2020 after the onset of the COVID-19 pandemic, reflecting institutional policy.

### 3.2. Content of SDM Dashboard

**Figure 2:**
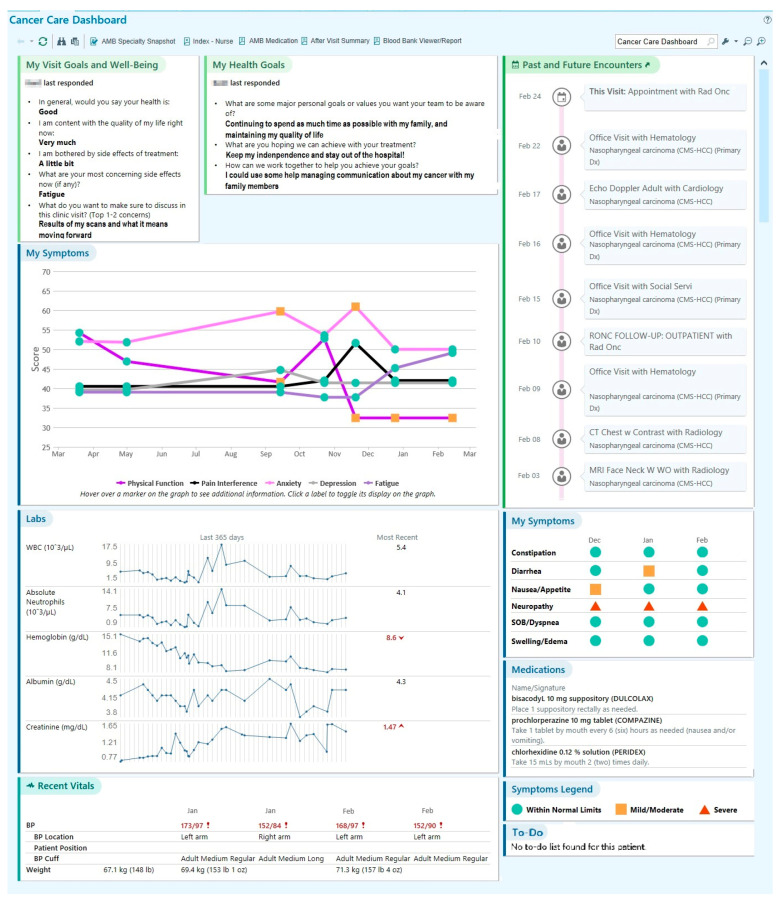
Cancer Care Dashboard. Reprinted with permission by © 2023 Epic Systems Corporation. MyChart^®^ is a registered trademark of Epic Systems Corporation. Information includes test patient and provider data only.

**Figure 3:**
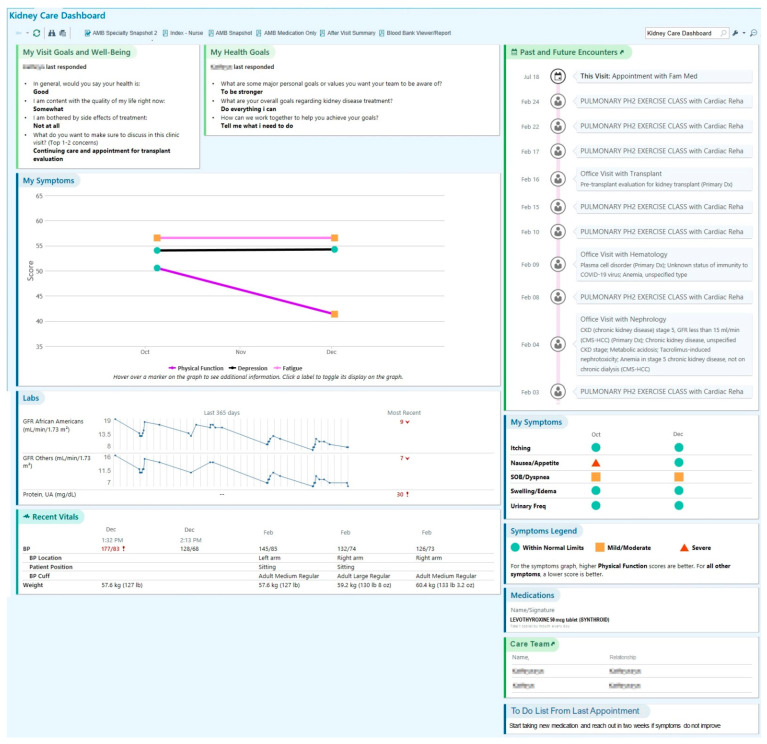
Kidney Care Dashboard. Reprinted with permission by © 2023 Epic Systems Corporation. MyChart^®^ is a registered trademark of Epic Systems Corporation. Information includes test patient and provider data only.

The co-design process resulted in two dashboards, each ready for clinical testing in either oncology or nephrology care (see Figure 2 and Figure 3 for de-identified screenshots of test patient data in the final dashboard displays). Across multiple co-design sessions, team members iteratively designed increasingly functional prototypes of the dashboard. First, co-design team members drafted individual mock-ups of their ideal dashboards, including data elements and presentation format. Then, the individual mock-ups were reviewed by the entire team to come to a mutual decision regarding the dashboard prototype; the dashboard prototype iteration process and outcomes are described in detail elsewhere [30]. To facilitate shared treatment planning between patients, care partners, and clinicians at the point of care, the final dashboard included sections to display patient-reported symptoms and goals; medication, laboratory, and vital signs data; upcoming appointments; and a “to-do” list (see Figure 2 and Figure 3). The dashboard was programmed to be individualized to each patient, pulling their unique PRO responses and other health-related information stored in the EHR to populate their dashboard before an upcoming visit.

### 3.3. Facilitators and Barriers of Implementing Co-Design Strategies and Adaptations

The respondents indicated that the fidelity and adoption of the co-design process were promoted by the strategy of inclusive team structure with varied stakeholders, collaborative team dynamics, and an iterative design process. Factors identified as barriers to fidelity included the time commitment involved, limited time for discussions during sessions, and desire for additional stakeholders representing intended end users (e.g., larger sample sizes of patient and care partners, as well as other clinicians from the respective disease groups) to be included on the teams. Challenges were also observed relating to the recruitment and retention of stakeholders, especially seriously ill patients and their caregivers. For example, we were never able to recruit a care partner for the kidney disease team. When needed, participants were identified by leveraging broader networks, such as one frontline clinician (dietician) employed outside of the local health system with significant professional experience who worked with our identified target patient population.

### 3.4. Co-Design Implementation Outcomes

The co-design implementation outcomes are summarized in Table 5.

#### 3.4.1. Fidelity and Adoption of Co-Design

Fidelity to the adoption of core co-design practices, measured by observer rubrics, demonstrated a mean of 19.1 out of 21 (SD = 1.63, range = 13 to 21). Self-reported adherence to the key principles of co-design was rated a mean of 18.5 out of 20 (SD = 1.4, range = 15 to 20) at the end of the co-design process. The mean collaboRATE score across sessions was 10.0 (standard deviation [SD] = 1.7, range = 4 to 12) (Table 5), demonstrating fidelity to the SDM process necessary for effective co-design in each co-design session. One-quarter (23%) of ratings across all sessions achieved the highest possible score (i.e., “Every effort was made” on all three items).

#### 3.4.2. Acceptability of Co-Design

In the final survey reflecting on the entire process, the mean collaboRATE score was 10.4 (SD = 1.4, range = 7 to 12), suggesting that the participants were satisfied with the degree of SDM that occurred during the co-design process overall. In addition, 29% of responses achieved the top box rating (i.e., “Every effort was made” on all three items).

#### 3.4.3. Feasibility of Co-Design

Scores for representation of stakeholders (reflecting feasibility and adoption of convening multistakeholder teams for the co-design process) were higher among patients with cancer than those with kidney disease (0.95 and 0.85 respectively). These scores indicate that, on average, 95% of stakeholder types were represented in cancer meetings and 85% were represented in CKD meetings.

#### 3.4.4. Effectiveness

Half (50%) of the respondents indicated that the process was fully effective in developing a dashboard designed to support SDM, with 50% responding as partially and 0% as not at all.

## 4. Discussion

We report on the successful implementation of a co-design strategy to build an SDM dashboard for healthcare service co-production. We followed CDIFM principles through deep stakeholder engagement to successfully co-design a dashboard to support SDM in patients with advanced cancer and chronic kidney disease. The co-design sessions achieved fidelity, acceptability, feasibility, adoption, and effectiveness for engaging a diverse group of stakeholders in a collaborative, respectful, and effective co-design process. The thoughtful assembly and ongoing engagement of co-design teams comprising an inclusive group of stakeholders (patients, care partners, clinicians, researchers, clinical quality personnel, and HIT) was imperative to the innovative capacity and overall success of this work. Applying an implementation research lens allowed us to understand the co-design process as it was implemented and to identify barriers that needed to be addressed through our strategies, such as creating subgroups in order for patients and care partners to have an equal voice. Our continuous collection of feedback from co-design team members after each session allowed us to adapt strategies throughout the process to better address barriers in the remainder of the upcoming sessions.

### 4.1. Evaluation of the Co-Design Process

The results of this paper demonstrate that our co-design process was implemented with fidelity and had high levels of acceptability, adoption, and perceived effectiveness. This paper outlines and confirms a process for linking principles of SDM with the co-design strategy of creating a clinical dashboard, which itself was designed to support SDM in healthcare encounters. Fidelity to SDM occurred during co-design, with team members reporting that co-design sessions involved a high degree of collaboration and effort made to understand stakeholders’ priorities for healthcare encounters and dashboard design. Based on observer rubrics and self-reports from team members, sessions also adhered to the evidence-informed co-design principles and practices [13,14,17,20,22,28] of including, respecting, engaging, and collaborating with all stakeholders while also being productive towards the end goal of dashboard development. These results indicate a successful implementation with good fidelity and adoption of co-design processes for the two clinical teams (advanced cancer and chronic kidney disease). Similarly, stakeholders were well-represented across working sessions, indicating the feasibility of convening a multi-stakeholder design team and adoption of co-design. Offering a remote option for participation was helpful in preventing stakeholder dropout and supporting accessibility for individuals not able to attend in person [31]. We believe that our continuous attention to and evaluation of the core principles and practices of co-design contributed to team members’ high degree of satisfaction with the degree of SDM in the co-design process (i.e., acceptability of co-design). The process was also effective in terms of developing the dashboards, with all respondents indicating that the final dashboard fully or partially met the intended objectives.

### 4.2. Co-Design Implementation Strategies and Learnings

Producing an effective and acceptable SDM dashboard tool required the implementation of important co-design strategies to achieve our five key principles of co-design. These included planning and supporting deep stakeholder engagement from a diverse group of patients, care partners, and clinicians, working in concert with experts in patient-reported outcome measurement science, implementation science, healthcare operations and quality measurement, and information technology. Cross-disciplinary dialogue helped teams work together to make decisions about what elements were essential versus desirable and helped them to make informed decisions about how to go about their iterative design processes to enhance the potential for long-term sustainability and scalability. For example, including HIT representation on these teams from the beginning allowed programmers to work with patients and clinicians to deeply understand their needs and facilitate authentic translation of ideas to a tangible end product, and to inform the team when ideas were not technically feasible in real time. It also inspired HIT to advance technical infrastructure in ways previously thought not to be possible. Thus, leveraging the collective expertise of these stakeholders was critical to the development of a feasible, flexible, and broadly applicable product.

Although co-design between researchers and stakeholders can enhance the implementation and future effectiveness of a new tool [32], published strategies and processes for conducting co-design activities and measuring the implementation outcomes are limited. There are not yet widely recognized best practices and drivers of effective co-design to help guide the approach and evaluation of this kind of work [31,33]. Our application of an implementation science lens offered the opportunity to measure and improve upon this complex process to meet specific shared goals. CDIFM steps 1 (define the problem) and 2 (establish context of use) were critical to cultivating a shared understanding of key contextual factors related to the intended purpose of the dashboard, the perspectives and experiences of end users, and the workflow demands of the clinical environment. CDIFM steps 3 (build consensus on design) and 4 (define and test specifications) were then essential to defining how the dashboard would be integrated into end users’ workflows and determining what foundational supports would be needed to facilitate its rollout and ongoing use. Our strategies and findings can help drive the successful implementation of a co-design process that includes PROs in the SDM context. Compared to traditional intervention design, co-design has both advantages and challenges. Among its advantages, engaging stakeholders in the design process of an intervention increases the likelihood that interventions will be acceptable by end users, which is necessary for future adoption and effectiveness of the intervention in practice [34,35]. End user input during the design phase is especially important for technology-enabled healthcare tools that rely on high levels of clinician and patient usability to be effective [36]. Research has also demonstrated that engaging in a participatory design process can have direct benefits for participants by increasing stakeholder knowledge and confidence in disease management [34]. Wider adoption and implementation of co-design may help to build a culture of patient engagement and co-production of their own health and healthcare [4,37]. However, this study also underlined challenges of co-design, most notably the time and resource constraints on maintaining stakeholder engagement in research and intervention development. Recruitment and retention of patients and care partners who are experiencing a serious illness can be difficult given the burden of disease, and research teams must be flexible and creative in coming up with solutions to accommodate these necessary stakeholders in order to achieve a representative sample of end users.

## 5. Limitations

The study has a number of limitations. We used a non-traditional application of collaboRATE surveys to capture SDM within a co-design process, and observers of the process may have been biased by their knowledge and attitudes toward the effort. Co-design work was conducted with a convenience sample in a well-resourced academic healthcare environment with a robust clinical research program, so the co-design process and outcomes described in this paper may not be generalizable to other settings. Due to the research topic of developing a digital dashboard for data visualization, there may have been a selection bias effect such that participating co-design members may have been younger or more technology-savvy compared to the intended end user population. Additionally, since measurement of effectiveness was limited to participants’ perceptions of whether intended objectives were met at the end of the co-design process, we will need to evaluate this again after full implementation. The present paper reports the successful implementation of a co-design process to create the two SDM clinical dashboards, but these results cannot be used to understand the dashboards’ usefulness in clinical practice. Future work evaluating their feasibility, adoption, acceptability, and effectiveness when implemented in routine practice in a follow-up study will be reported in subsequent journal articles.

## 6. Implications and Future Directions

Our successful co-design of the SDM dashboards has potential to improve quality of care and quality of life for patients with advanced cancer and CKD. Patients with advanced cancer and CKD often have a high burden of disease, including debilitating symptoms and increased healthcare encounters that can be financially costly and further disrupt quality of life [24,25]. Therefore, patients may benefit from routine PRO monitoring and feedback of PRO data to clinicians through a visual dashboard to support SDM and align care with patients’ preferences and health status. Future research from this co-design work will examine the potential implementation strategies, outcomes, and effectiveness of using the SDM clinical dashboard in the flow of clinical practice for advanced cancer and CKD. Key effectiveness outcomes that will be examined include whether using the dashboard can improve SDM in clinical encounters, symptom severity, and other PROs and reduce patient utilization of the ED, ICU, and other costly healthcare services. The results of the pilot study are pending and will be reported in future peer-reviewed journal articles.

Future research should also examine possible intersections between our SDM dashboard and recent advances in artificial intelligence (AI). AI techniques, such as natural language processing and machine learning algorithms, enable the real-time analysis of PROs and other critical data, which may be able to provide more personalized and timely prognostic predictions. For example, a recently developed AI model was developed to use electronic PRO data to predict the risk of adverse events from cancer immune checkpoint inhibitor therapies [38]. AI-enabled prediction tools such as this could be incorporated into the SDM dashboard to enhance clinical decision making. For instance, when combined with clinical judgement from a clinician, AI models may be able to facilitate acceptable interventions to anticipate and intervene with adverse outcomes early on [39]. In turn, routine implementation of clinical dashboards such as our co-designed SDM dashboard can support the continuous re-evaluation and validation of deep learning models to improve their accuracy over time. A key challenge of embedding PROs into AI-enabled health technologies is the lack of access to large databases of PRO data that are representative of the health system [40]. Our SDM dashboard is intended to facilitate a learning health system by routinely collecting PROs in multiple patient populations over time to continuously inform healthcare system practice and policy [11].

## 7. Conclusions

In the present study, a co-design process was applied to develop a tool intended to promote SDM for co-production of healthcare services in advanced cancer and CKD. The results found that commitment and collaboration of an appropriately diverse group of stakeholders and end users working in concert to envision, build, and plan for implementation was critical to this process. We were able to successfully implement the codesign process despite challenges due to COVID-19, with high ratings for implementation outcomes, including fidelity and adoption of co-design practices and high degrees of SDM that occurred during the co-design process. These results contributed to the creation of the dashboard, which incorporated multiple stakeholder views and priorities to meet its intended objective. Documentation of the specific steps and implementation outcomes of co-design in this article will help to support the adoption of similar work in the future and of SDM in practice. The results of the pilot study of the SDM dashboards in clinical practice will provide further opportunities to evaluate the impact of the co-designed tools.

## Figures and Tables

**Table 1:** Characteristics of co-design teams displayed as N (%).

	Cancer Team (N = 25)	CKD Team (N = 24)	Total (N = 35)
Gender			
Female	20 (80%)	14 (58.3%)	24 (68.6%)
Male	5 (20%)	10 (41.7%)	11 (31.4%)
Role			
Patient	2 (8%)	3 (12.5%)	5 (14.3%)
Care partner	2 (8%)	0 (0%)	2 (5.7%)
Physician	3 (12%)	3 (12.5%)	6 (17.1%)
Physician Assistant *	1 (4%)	1 (4.2%)	2 (5.7%)
Nurse Practitioner *	2 (8%)	0 (0%)	2 (5.7%)
Nurse *	0 (0%)	1 (4.2%)	1 (2.9%)
Social Worker *	1 (4%)	1 (4.2%)	2 (5.7%)
Clinical Psychologist **	3 (12%)	3 (12.5%)	3 (8.6%)
Dietician *	0 (0%)	1 (4.2%)	1 (2.9%)
Health Informaticist	3 (12%)	3 (12.5%)	3 (8.6%)
Health System Quality Leader **	4 (16%)	4 (16.7%)	4 (11.4%)
Researcher **	4 (16%)	4 (16.7%)	4 (11.4%)

Note. Cancer and CKD teams were not mutually exclusive; there was some overlap among cancer and CKD team members because all participants who fell into the following roles participated in both groups: clinical psychologist, health informaticist, health system quality leader, and researcher. * Clinician types that are sometimes referenced in aggregate as “frontline clinicians”. ** Professional specialties/roles of “facilitators” and/or “project team members”.

**Table 2:** Outline of Northwestern shared decision making dashboard co-design activity (modified from Perry et al. (2022) [26], which was published (and can be reproduced) under the terms of Creative Commons Attribution 4.0 license).

Activity	Co-Design Phase	Objectives	Outcomes	CDIFM Phase (1–4)
**Co-design Launch Meeting**	Introduction	Introduction to the project and key concepts Review of co-design working structure and scope of work	Baseline knowledge of SDM and co-design Understanding of co-design participation expectations	1
**Working Session #1**	Establish Shared Priorities	Team building Identify facilitators and barriers to SDM and health coproduction Explore how better sharing of information can help	Defined barriers and facilitators to patient–clinician communication in optimal care planning List of ideas on how actionable information supports improved communication and delivery of care	1, 2
**Working Session #2**	Establish Shared Priorities	Develop an understanding of how information can address facilitators and barriers to improving care management	Advancement of ideas and shared priorities	2
**Working Session #3**	Establish Shared Priorities	Identify common themes, priorities, and values on information and SDM of dashboard end users Develop team-specific co-design objectives	Establish priority of information sources critical to SDM and communication Draft team-specific objectives	2, 3
**Working Session #4**	Envision Dashboard	Refine team-specific objectives Introduce options for data elements to populate dashboards Envision dashboard	Final team objectives Defined preliminary options for data elements to populate dashboards Dashboard sketches	1, 2, 3
**Working Session #5**	Draft initial Specifications	Explore use of a dashboard in a case example (persona) to advance emerging dashboard concepts	Critical thinking on application of initially proposed dashboard specifications Validation of critical data features and functionalities Suggested revisions to draft dashboards	2, 3, 4
**Working Session #6**	Evaluate and Test	Explore use of a dashboard in a case example (persona) focused on a point of SDM to advance emerging dashboard concepts	Critical thinking on application of initially proposed dashboard specifications Validation of critical data features and functionalities Suggestions for revisions to draft dashboards	2, 3, 4
**Working Session #7**	Evaluate and Test	Define priority dashboard elements by dashboard user type Propose questions for external validation (focus groups) Exchange ideas and plans for dashboard concepts between cancer and kidney disease teams	Reactions and requested revisions to latest iteration of the dashboards Define priorities by stakeholder types and disease-specific teams to ensure shared utility	2, 3
**Working Session #8**	Implementation Planning	Review dashboard drafts and confirm alignment with co-design teams’ visions Review data sources and measures to populate dashboards	Validation and editing of draft dashboard(s) Stakeholder-specific perspectives contributed to inform implementation plan	2, 3
**Working Session #9**	Implementation Planning	Review feasible dashboard display options Confirm completeness and appropriateness of planned data elements	Validation and editing of draft dashboard(s) Confirm data elements to populate dashboard(s)	2, 3
**Working Session #10**	Implementation Planning	Demo of programmed dashboard display Cancer and kidney co-design teams present respective dashboards and exchange ideas	Solidify dashboard core of common sections/layout Confirm need and ability to maintain flexibility to customize data elements to populate dashboard sections	2, 3
**Working Session #11**	Implementation Planning	Demo and critical review of fully programmed dashboards	Validation and editing of draft dashboard(s)	3, 4
**Physician Champion Dashboard Implementation Survey**	Implementation Planning	Establish clinic-specific needs/preferences for dashboard data elements and technical functionality of support pathways	Confirm disease-specific customizations Confirm patient criteria for automated delivery of questionnaires to populate the dashboard	Not a CDIFM Phase
**Working Session #12**	Implementation Planning	Interactive demo of fully programmed dashboards and questionnaires Establish specifications for alerts (symptom thresholds and routing) Determine communication strategy and framing of patient-facing questionnaires	Orientation to dashboard in live environment Define mechanics of implementation for patient and clinician dashboard users	2, 3
**Working Session #13 (Physician Champion Working Meeting)**	Implementation Planning	Confirm final dashboard specifications Demonstration and discussion of in-basket alerts Confirm final patient dashboard user criteria Confirm implementation workflows	Define alert pathways and thresholds Finalize dashboard specifications Finalize feasible patient dashboard user criteria Finalize Implementation Workflows	2, 3, 4
**Co-design Wrap Up** **Meeting**	Reflect & Transition	Live demo of the pre-final dashboards and questionnaires Conduct a reflection on the entire coproduction process with respect to participation in co-design activities Examine and discuss implications of COVID-19 and considerations for telehealth.	Final validation of dashboard specifications Reflections on the dashboard coproduction process and experience Initial considerations of potential effect of COVID-19	4

**Table 3:** Mapping of Use of implementation strategies for co-design to achieve five key principles. X notes that the strategy targeted the key principle.

Strategy	Inclusivity	Respect	Participation	Iteration	Outcomes Focused
Stakeholder feedback on evolving dashboard prototype		X	X	X	X
Inclusion of HIT	X		X	X	X
Design of sessions to accommodate stakeholders	X	X	X		
Establish principles of equity in contribution of ideas	X	X	X		
Establish agreed on goals of the dashboard					X
External consultation from Dartmouth			X		X
Revisiting of CDFIM concepts throughout process			X		X
Incorporation of virtual participation options	X	X	X		

**Table 4:** Outcome measures and data sources.

Domain(s)	Description	Scoring Metric	Assessment Point(s)	Data Sources
Fidelity: the degree to which an intervention is implemented as planned	Three items measuring the perceived degree to which the following occurred **during each co-design session** met the criteria for SDM as part of co-design (from 0 to 4): (1) How much effort was made to understand the health issues which you think should be addressed by shared decision making? (2) How much effort was made to listen to the things that matter most to you in the discussion about the design and choosing what to do next? (3) How much did team members actively collaborate during this discussion?	**Primary:** Total scores of 0 to 12 (low SDM to high SDM) **Secondary:** Presence/absence of “Top Box” score (a 12 out of 12 on the total score)	Administered after each co-design session	Adapted CollaboRATE measure
Acceptability: the degree to which users find the intervention (co-design) satisfactory, agreeable	Three items measuring the perceived degree to which the following occurred during co-design process **as a whole** met the criteria for SDM as part of co-design (from 0 to 4): (1) How much effort was made to understand the health issues which you think should be addressed by SDM? (2) How much effort was made to listen to the things that matter most to you in the discussion about the design and choosing what to do next? (3) How much did team members actively collaborate during the sessions?	**Primary:** Total scores of 0 to 12 (low SDM to high SDM) **Secondary:** Presence/absence of “Top Box” score (a 12 out of 12 on the total score)	Administered after the last co-design session in a post-co-design reflection survey	Adapted CollaboRATE measure
Fidelity: degree to which 7 practices of codesign were met Adoption: Participation in codesign	7 practices, rated from 1 (absent) to 3 (fulfilled): (1) All experiences and perspectives are (treated as) equally valuable (2) All voices are needed (3) Listen to what others are saying (4) Ask questions (5) Be constructively critical (6) Actively collaborate with fellow team members (7) Be respectful of privacy and confidentiality	Total scores of 7 to 21	Rated at each co-design session by outside observers	Observer-rated rubrics of co-design practices
Fidelity: degree to which the 5 core principles of codesign were met	5 key principles of co-design: (1) Inclusive (2) Respectful (3) Participative (4) Iterative (5) Outcomes-focused Also included open-ended questions on barriers and areas for improvement	Scores from 5 to 20	Administered after the last co-design session in a post-co-design reflection survey	Co-design participant-rated surveys of principles of co-design
Feasibility: ability of stakeholders to participate in the co-design process Adoption: actual participation of the stakeholder on the co-design process	Includes documentation of stakeholder representation and meeting modality across all	Mean scores of stakeholder representation from 0 to 1, indicating the proportion of stakeholder types represented across the co-design sessions. Frequency of attendance modalities from 0% to 100%	Calculated after the co-design process	Co-design session attendance logs (Supplementary Materials File S1)
Effectiveness: degree to which the co-design process resulted in a dashboard designed to meet the project goals	Poll of perceptions on whether the dashboard objectives were met after session 13.	Proportion of respondents selecting each response option (yes, partially, no) from 0% to 100%	Administered after the last co-design session in a post-co-design reflection survey	Co-design effectiveness poll

**Table 5:** Metrics of fidelity, acceptability, adoption, feasibility, and Effectiveness.

Implementation Outcome Measure	Respondents/Data Sources	Metric	Observed Range	Mean	Standard Deviation
Fidelity by session	Co-design team members	Adapted collaboRATE (see Table 4; Score 0–12)	4–12	10.0	1.7
Fidelity, adoption by session	Observers	Observer Rubric (Score 7–21)	13–21	19.1	1.6
Fidelity, rated after co-design process was complete	Co-design team members	Key principles of co-design (Score 5–20)	15–20	18.5	1.4
Acceptability, rated after co-design process was complete	Co-design team members	Adapted collaboRATE (see Table 4; Score 0–12)	7–12	10.4	1.4
Feasibility, adoption	Program data	Attendee representation from 0 to 1 (combined groups)	0.71–1.00	0.90	0.07
		Attendee representation from 0 to 1 (cancer group)	0.71–1.00	0.95	0.09
		Attendee representation from 0 to 1 (kidney disease group)	0.71–1.00	0.85	0.07
		Frequency of sessions with live or remote participation	**Group**	**Live**	**Remote**
			Cancer	14/15 (93%)	9/15 (60%)
			Kidney Disease	14/15 (93%)	14/15 (93%)
Effectiveness	“Does the dashboard meet the intended (group defined) objective?”	Yes, partially, no	N = 10 Yes 50% (5) Partially 50% (5) No 0% (0)		

## Data Availability

The datasets used and/or analyzed during the current study are available from the corresponding author upon reasonable request.

## Supplementary Materials

The following supporting information can be downloaded at: https://www.mdpi.com/article/10.3390/jcm13144178/s1. Supplementary Materials File S1. Stakeholder Representation & Meeting Modality Across Co-design Activity. Supplementary Materials File S2. Observer Rubric.

## Abbreviations

APP	Advanced Practice Professional (e.g., nurse practitioner, physician assistant)
CDIFM	Coproduction Design and Implementation Flow Model
CKD	Chronic Kidney Disease
EBP	Evidence-Based Practice
EHR	Electronic Health Record
GI	Gastrointestinal
HIT	Health Information Technology
IT	Information Technology
NM	Northwestern Medicine
NU	Northwestern University
PRO	Patient Reported Outcomes
PROM	Patient Report Outcome Measure
PROMIS	Patient Reported Outcome Measurement Information System
SDM	Shared decision making
