# Supplementary material for: Implementation of a Co-Design Strategy to Develop a Dashboard to Support Shared Decision Making in Advanced Cancer and Chronic Kidney Disease
Source: J Clin Med. 2024 Jul 17;13(14):4178. doi: 10.3390/jcm13144178 (PMC11278116; doi:10.3390/jcm13144178)
Supplement: Supplementary file 1 [file jcm-13-04178-s001.zip › Dashboard coproduction_additional file_7.10.24vm.pdf]

Supplementary Materials File S1. Stakeholder Representation & Meeting Modality Across Co-design Activity

[Data extracted from session attendance logs and project records]

	Combined Summary (Cancer & Kidney Disease)		
	Representation	Meeting Modality	
	Proportion of Stakeholder Types Represented	Live Participation	Remote Participation
Co-design Launch	**0.93**	Y	Y
Working Session #1	**0.71**	Y	Y
Working Session #2	**0.93**	Y	Y
Working Session #3	**0.93**	Y	Y
Working Session #4	**0.93**	Y	Y
Working Session #5	**0.93**	Y	Y
Working Session #6	**0.93**	Y	Y
Working Session #7	**0.93**	Y	Y
Working Session #8	**0.79**	Y	Y
Working Session #9	**0.93**	Y	Y
Working Session #10	**0.93**	Y	Y
Working Session #11	**0.86**	Y	Y
Working Session #12	**0.86**	Y	
Working Session #13 (Physician Champion Working Meeting)	**1.00**	Y	N
Co-design Wrap Up Meeting	**0.93**	N	Y
Frequency/Total number of applicable sessions		**14/15 (93%)**	**14/15 (93%)**
Mean Score Across all Sessions	**0.90**	0.93	0.93

Across all sessions overall attendance ranged from 2-5 among patients, 1-2 among care partners, 3-7 among frontline clinicians, 2-5 among physician champions, 0-2 for HIT professionals, and 4-10 for facilitators (4-8 for projectteam members and 0-2 for quality leaders).

	Kidney Disease										
	Stakeholder Type							Representation		Meeting Modality	
	Patient	Care Partner	Frontline Clinician	Physician Champion	Health IT	NM Quality	NU (Northwestern University) Researcher	Score (0-7)	Proportion of Stakeholder Types Represented	Live Participation	Remote Participation
Co-design Launch	Y	N	Y	Y	Y	Y	Y	6	0.86	Y	Y
Working Session #1	Y	N	Y	Y	N	Y	Y	5	0.71	Y	Y
Working Session #2	Y	N	Y	Y	Y	Y	Y	6	0.86	Y	Y
Working Session #3	Y	N	Y	Y	Y	Y	Y	6	0.86	Y	Y
Working Session #4	Y	N	Y	Y	Y	Y	Y	6	0.86	Y	Y
Working Session #5	Y	N	Y	Y	Y	Y	Y	6	0.86	Y	Y
Working Session #6	Y	N	Y	Y	Y	Y	Y	6	0.86	Y	Y
Working Session #7	Y	N	Y	Y	Y	Y	Y	6	0.86	Y	Y
Working Session #8	Y	N	Y	Y	Y	N	Y	5	0.71	Y	Y
Working Session #9	Y	N	Y	Y	Y	Y	Y	6	0.86	Y	Y
Working Session #10	Y	N	Y	Y	Y	Y	Y	6	0.86	Y	Y
Working Session #11	Y	N	Y	Y	Y	Y	Y	6	0.86	Y	Y
Working Session #12	Y	N	Y	Y	Y	Y	Y	6	0.86	Y	Y
Working Session #13 (Physician Champion Working Meeting)	N/A	N/A	N/A	Y	Y	Y	Y	4	1.00	Y	N
Co-design Wrap Up Meeting	Y	N	Y	Y	Y	Y	Y	6	0.86	N	Y
Proportion of Sessions	14/14 (100%)	0/14 (0%)	14/14 (100%)	15/15 (100%)	14/15 (93%)	14/15 (93%)	15/15 (100%)			**14/15 (93%)**	**14/15 (93%)**
Mean Score Across all Sessions	1.00	0.00	1.00	1.00	0.93	0.93	1.00		**0.85**		

	Cancer										
	Stakeholder Type							Representation		Meeting Modality	
	Patient	Care Partner	Frontline Clinician	Physician Champion	Health IT	NM Quality	NU (Northwestern University) Researcher	Score (0-7)	Proportion of Stakeholder Types Represented	Live Participation	Remote Participation
Co-design Launch	Y	Y	Y	Y	Y	Y	Y	7	1.00	Y	Y
Working Session #1	Y	Y	Y	N	N	Y	Y	5	0.71	Y	N
Working Session #2	Y	Y	Y	Y	Y	Y	Y	7	1.00	Y	Y
Working Session #3	Y	Y	Y	Y	Y	Y	Y	7	1.00	Y	N
Working Session #4	Y	Y	Y	Y	Y	Y	Y	7	1.00	Y	Y
Working Session #5	Y	Y	Y	Y	Y	Y	Y	7	1.00	Y	N
Working Session #6	Y	Y	Y	Y	Y	Y	Y	7	1.00	Y	Y
Working Session #7	Y	Y	Y	Y	Y	Y	Y	7	1.00	Y	Y
Working Session #8	Y	Y	Y	Y	Y	N	Y	6	0.86	Y	N
Working Session #9	Y	Y	Y	Y	Y	Y	Y	7	1.00	Y	Y
Working Session #10	Y	Y	Y	Y	Y	Y	Y	7	1.00	Y	Y
Working Session #11	Y	Y	Y	N	Y	Y	Y	6	0.86	Y	N
Working Session #12	Y	Y	Y	N	Y	Y	Y	6	0.86	Y	Y

Working Session #13 (Physician Champion Working Meeting)	N/A	N/A	N/A	Y	Y	Y	Y	4	1.00	Y	N
Co-design Wrap Up Meeting	Y	Y	Y	Y	Y	Y	Y	7	1.00	N	Y
Proportion of Sessions	14/14 (100%)	14/14 (100%)	14/14 (100%)	12/15 (80%)	14/15 (93%)	14/15 (93%)	15/15 (100%)			**14/15 (93%)**	**9/15 (60%)**
Mean Score Across all Sessions	1.00	1.00	1.00	0.80	0.93	0.93	1.00		**0.95**		
