# Supplementary material for: Implementation of a Co-Design Strategy to Develop a Dashboard to Support Shared Decision Making in Advanced Cancer and Chronic Kidney Disease
Source: J Clin Med. 2024 Jul 17;13(14):4178. doi: 10.3390/jcm13144178 (PMC11278116; doi:10.3390/jcm13144178)
Supplement: Supplementary file 1 [file jcm-13-04178-s001.zip › Dashboard coproduction_additional file2_7.10.24vm.pdf]

Supplementary Materials File S2. Observer Rubric

ASSESSMENT OF FIDELITY TO CODESIGN PRINCIPLES & NORMS

Date: ______________________

Codesign Working Session #_____

Codesign Team: ______________

Initials: _____________________

Score:

Instructions:

Based on your observations, circle the response (columns 1-3) for each core practice that best describes the dynamics of the group during this session. Add up the total number of points and record in the score above.

Core practices	1	2	3
All experiences and perspectives are (treated as) equally valuable	Disagree- some people's perspectives are generally ignored or not included	Most people's perspectives are included and discussed but not always	Agree completely- All are included in discussions
All voices are needed	The facilitator does not encourage and ensure everyone participates	The facilitator encourages participation, but there are some people who speak up infrequently or not at all	The facilitator makes sure that everyone speaks up and participates
Listen to what other are saying	Interruptions are common especially for some participants	Some minimal but most or all are respectful disruptions.	People listen to each other all the time (no interruptions)
Ask questions	Only a few people ask most of the questions	Questions are asked by some (one half or more) of the group but not all.	Everyone asks questions
Be constructively critical	Criticism is generally negative	Little to no criticism is shared	Criticism is generally constructive
Actively collaborate with fellow team member	None or very little collaboration seen in codesign and discussions between team members	Some collaboration is seen in codesign process and discussion between team members but less than 1/2 time	Collaboration is generally or always seen in codesign process and discussions between team members
Be respectful of privacy and confidentiality	Privacy and confidentiality is not respected	Some lapses are seen but they are rare (once per session at most)	Privacy and confidentiality is always respected
